# Lynch Syndrome-Associated Endometrial Cancer With Combined EPCAM-MSH2 Deletion: A Case Report

**DOI:** 10.3389/fonc.2022.856452

**Published:** 2022-03-04

**Authors:** Rong Huang, Xiangyu Deng, Zhenhua Zhang, Qinglian Wen, Dan Li

**Affiliations:** Department of Oncology, Affiliated Hospital of Southwest Medical University, Luzhou, China

**Keywords:** lynch syndrome, endometrial carcinoma, MSH2, EPCAM, case

## Abstract

**Background:**

Lynch syndrome (LS), an autosomal dominant disorder, is characterized by germline pathogenic variants in DNA mismatch repair (MMR) genes like MSH2. EPCAM deletions cause a minority (3%) of LS cases. However, there are only a few reports of LS-associated endometrial cancer (LS-EC) induced by the inactivation of the MSH2 gene due to EPCAM deletions.

**Case Presentation:**

We present the case of a 45-years old woman diagnosed with endometrial cancer (EC). Definitive surgery revealed meso-differentiated endometrioid adenocarcinoma, stage IA without lymph-vascular space invasion. Four months later, she received radiation therapy (^125^I radioactive seeds implantation), and platinum-containing regimen combined chemotherapy because of vaginal stump metastasis of EC. After five years, we performed immunohistochemistry (IHC) on pelvic mass because of presacral metastatic lymph node. IHC showed the absence of MSH2 and MSH6 protein expression in the pelvic mass tissue. Peripheral blood was used for genetic testing based on her cancer diagnosis and family history of cancer in close relatives. Genetic testing revealed deletions of exon 8 and 9 in EPCAM and deletions of exon 1 and 8 in MSH2; thus, we diagnosed the presence of LS. The patient underwent interstitial brachytherapy (BT) of the presacral metastatic lymph node.

**Conclusion:**

This case highlights that patients with LS-EC who are carriers of combined EPCAM-MSH2 deletion might experience better oncologic outcomes even with early recurrence.

## Introduction

Lynch syndrome (LS) is caused by germline pathogenic variants (PVs) in one of the DNA mismatch repair (MMR) genes (MLH1, MSH2, MSH6, or PMS2) or by deletions in the epithelial cell adhesion molecule gene (EPCAM), which increases susceptibility to colorectal and endometrial cancers and other tumors (1–3). There is evidence that cancer risk depends on the affected gene (4, 5). MLH1 and MSH2 are most commonly associated with a higher risk of colorectal cancer. And female MSH6 carriers may have the highest risk of endometrial cancer (EC). Up to 3% of LS cases are due to variants involving the 3’ end of the EPCAM gene (immediately adjacent to MSH2), which result in hypermethylation of the MSH2 promoter or partial deletion of MSH2 (6, 7). EPCAM deletions cause LS by causing transcriptional readthrough into the neighboring gene, MSH2, leading to its epigenetic silencing. The difference in tumor occurrence or spectrum resulting from MSH2 mutation carriers *versus* EPCAM deletion carriers may be related to the mosaic pattern of MSH2 inactivation displayed by EPCAM deletion carriers (8, 9). Unlike colorectal cancer where EPCAM deletions are an independent risk factor, EPCAM deletions will increase the risk of EC only if deletions extend near the MSH2 promoter (10). The cumulative risk by the age of 70 years of EC in women with EPCAM deletions was 12% and that of colorectal cancer in EPCAM deletion carriers was 75%. The risk of developing colorectal cancer was not significantly different between EPCAM deletion carriers and EPCAM-MSH2 deletion (*p*=0·8609) or MSH2 mutation (*p*=0·5892) carriers. However, the risk of developing EC was lower in EPCAM deletion carriers than in EPCAM-MSH2 deletion (*p*<0·0001) or MSH2 mutation (*p*=0·0006) carriers (10).

This study shows the presentation and outcomes of a patient with LS-EC with combined EPCAM-MSH2 deletion.

## Case Presentation

The patient is a 45-years-old Chinese woman who visited Yunnan Cancer Hospital and complained of prolonged and increased menstruation. Her past medical history was unremarkable. After an EC diagnosis, the gynecologist performed a total hysterectomy, bilateral salpingo-oophorectomy, and pelvic lymph node dissection under general anesthesia. Histopathological examination of surgically obtained samples led to a meso-differentiated endometrioid adenocarcinoma diagnosis. Pathological staging of surgery identified the disease as stage IA with no lymph-vascular space invasion. Four months after the operation, colonoscopy and computed tomography (CT) scan showed rectal occupancy. She underwent exploratory laparotomy and was diagnosed with vaginal stump metastasis of EC. As the patient refused to have an enterostomy, the radiation oncologist performed radiation therapy (^125^I radioactive seeds implantation), followed by six cycles of chemotherapy (paclitaxel combined with carboplatin). Re-examination of imaging data showed that the lesion was in complete remission. During the 5-year follow-up period, there were no reports of the recurrence of gynecological tumors.

**Figure 1:**
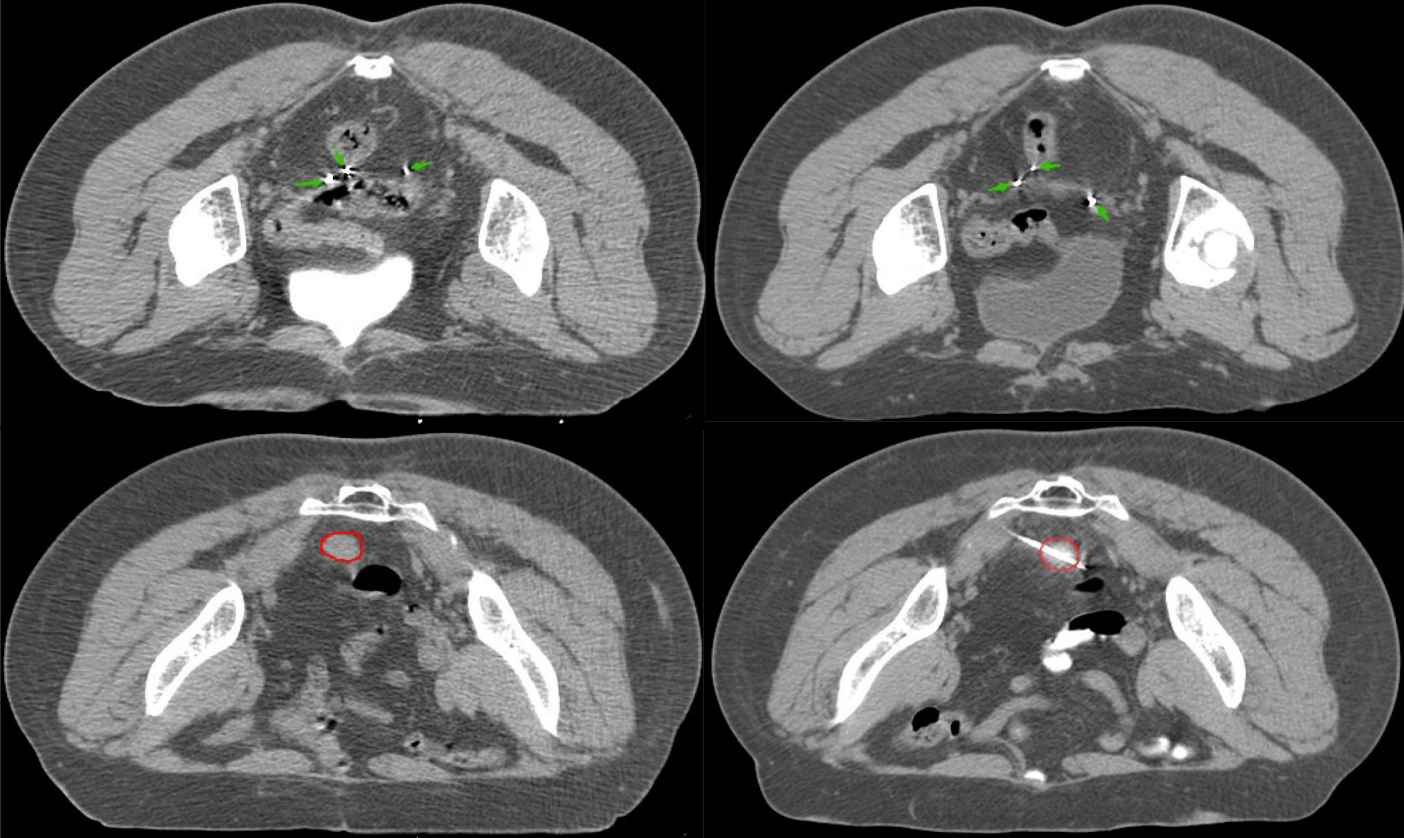
There are twenty radioactive seeds in CT images of pelvic recurrence. The green arrow represents ^125^I radioactive seed; the red circle represents lesions.

**Figure 2:**
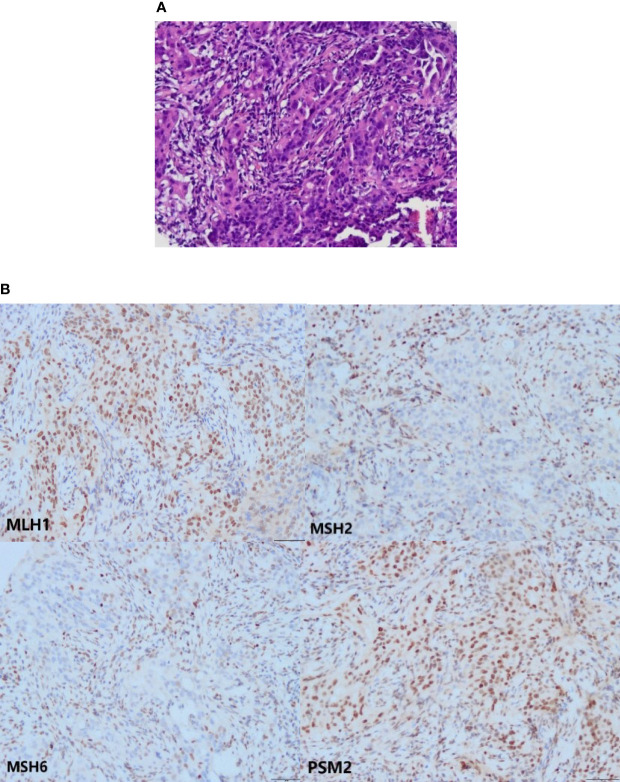
The HE and IHC staining. **(A)** The HE staining of pelvic mass tissues (the microscope magnifying×200). **(B)** Loss of MSH2 and MSH6 proteins in the tumor cells. Expression of MLH1 and PSM2 proteins in the tumor cells (the microscope magnifying×200).

**Figure 3:**
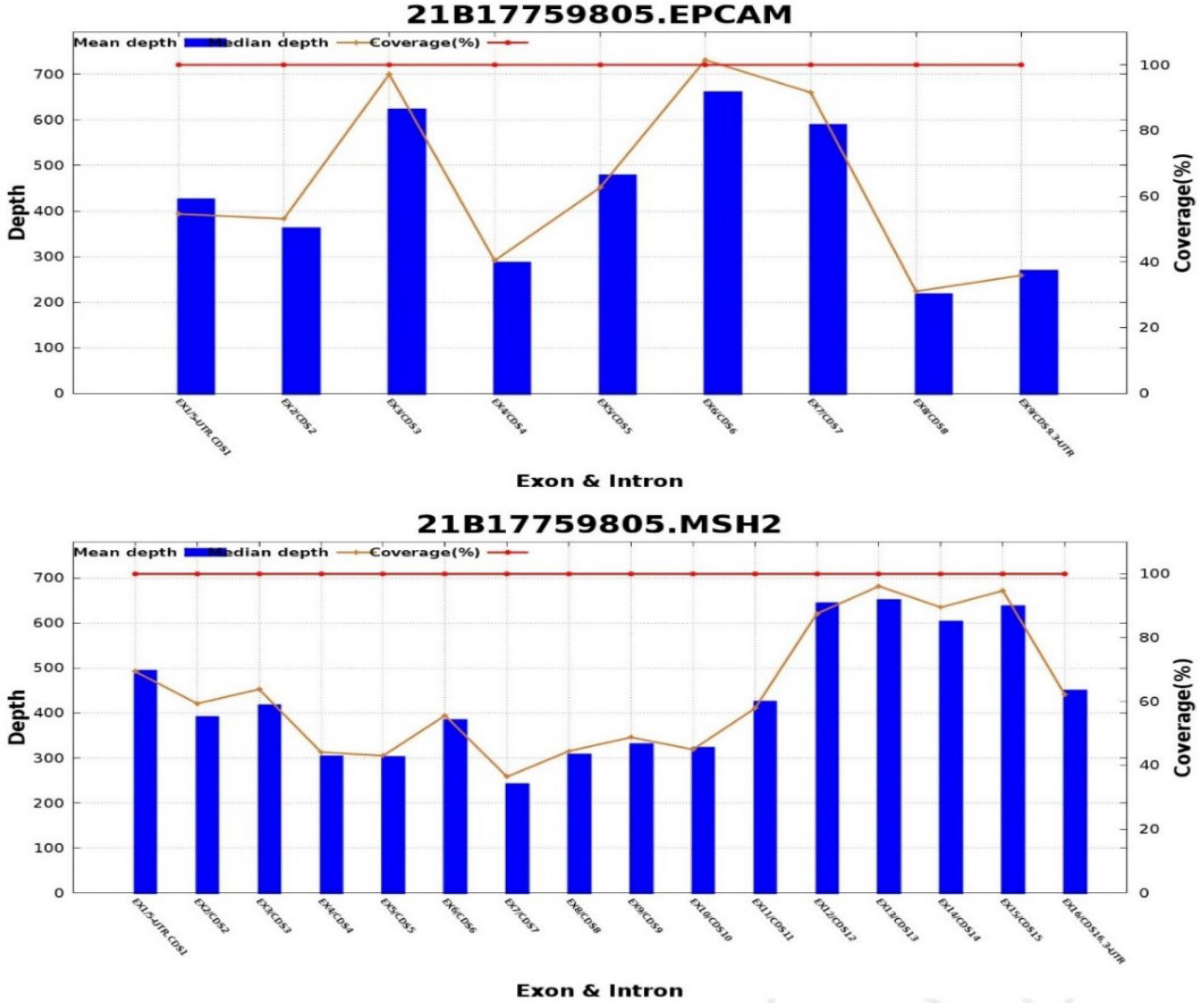
The results of genetic analysis.

**Figure 4:**
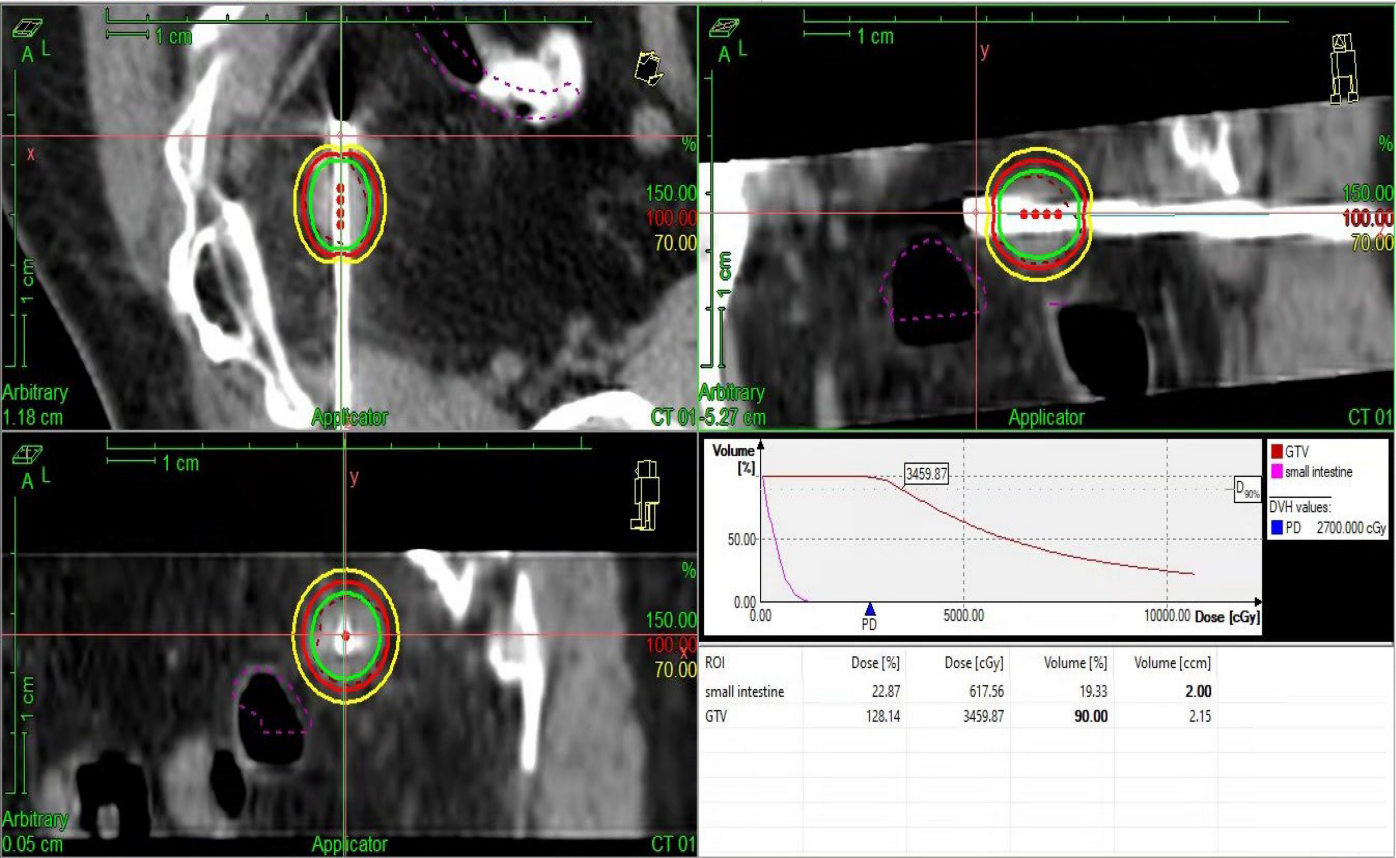
Three-dimensional conformal dose assessment for interstitial BT.

Five years later, she visited our hospital with a pelvic mass during follow-up (**Figure 1**). **Figure 2** shows hematoxylin-eosin (HE) staining and IHC staining of pelvic mass tissues. IHC staining results: CK7(+), CK20 (-), CEA (+), Vim (-), ER (-), PR (+, 60%), P16(+), Ki67 (+, 50%), P53 (+, 40%), PMS2 (+), MLH1 (+), MSH6(-), MSH2 (-). The patient had two first-degree relatives with endometrial and colorectal cancer and two second-degree relatives with gallbladder and stomach cancer. We provided her with genetic counseling based on her cancer diagnosis and family history of multiple cancers. After obtaining consent, we carried out the genetic analysis of her peripheral blood. Results were as follows: deletions of exon 8 and 9 in EPCAM and deletions of exon 1 and 8 in MSH2 (**Figure 3**); thus, we diagnosed the presence of LS. At the same time, the radiation oncologist of our hospital performed interstitial BT of presacral metastatic lymph node, and the prescription dose was 2700cGy. **Figure 4** shows three-dimensional conformal dose assessment for interstitial BT.

## Discussion

Approximately 3% of endometrial cancers are causally related to PVs in one of the DNA MMR genes (MLH1, MSH2, MSH6, and PMS2) (11). Among first cancer detected in each patient from the first prospective lynch syndrome database, the EC cumulative incidences at 70 years by gene were 34%, 51%, 49% and 24% for MLH1, MSH2, MSH6 and PMS2 mutation carriers, respectively (5). EPCAM is not an MMR gene, but structural alterations in EPCAM may lead to LS as it is adjacent to the MSH2 gene (9). In LS, the deletion of heterozygote sequences at the 3’ end of EPCAM can lead to the inactivation of MSH2 in tissues expressing EPCAM due to its promoter hypermethylation (12). And what’s more, the EPCAM 3’ end deletion might extend to the first MSH2 exon, which includes the promoter region, resulting in the suppression of both EPCAM protein and MSH2 protein expressions without MSH2 hypermethylation (7). Therefore, MSH2 negative patients need to be tested for EPCAM deletions. This patient’s IHC showed the absence of MSH2 and MSH6 protein expression in the tumor cells. According to the recommendation of the Manchester International Consensus Group, patients with MSH2, MSH6 or PMS2 protein deletion require germline testing related to LS (13). This report is a kind of MSH2 germline mutation EC with deletions of EPCAM, a relatively rare condition.

A related study showed that the risk of EC in EPCAM deletion carriers might depend on the location and size of the deletion. This cumulative risk of developing EC in women with EPCAM deletions at age 70 (12%) was significantly lower than in EPCAM-MSH2 deletion (51%) or MSH2 mutation (55%) carriers (*p*<0·0001, *p*=0·0006, respectively) (10). Ring et al. found mutations associated with LS in 5.8% of 381 EC cases. For mutations in MLH1, MSH2, and EPCAM-MSH2, 80% of patients were diagnosed with EC at <50 years of age, and most patients had a family history of LS-associated cancer (14). The patient’s age and family history in this report are consistent with the characteristics of EPCAM-MSH2 mutation.

Molecular tumor screening for LS in EC was supported by the Society of Gynecologic Oncology clinical practice statement in 2017. However, the prognostic value of MMR status has not been fully established in EC outside the LS diagnosis. Data from studies show that MMR deficiency is associated with lower recurrence rates, better progression-free, and overall survival in advanced EC treated with adjuvant therapy (15, 16). Contrastingly, MMR deficiency in early-stage EC is associated with poor prognostic factors and worse progression-free survival (17, 18). Moreover, some studies have shown that MMR deficiency in LS-EC could be targeted for immunotherapy (19, 20). Pembrolizumab and nivolumab have been recommended by the NCCN guidelines for advanced or recurrent EC patients with MMR deficiency (21). The patient with LS-EC in stage IA, carrier of combined EPCAM-MSH2 deletion, soon developed a local pelvic recurrence and could not achieve satisfactory surgical resection. However, local ^125^I radioactive seeds implantation and platinum-containing regimen combined chemotherapy led to a five-year disease-free survival period.

After five years, the presacral metastatic lymph node was identified as endometrioid adenocarcinoma by biopsy and IHC. Even though the recurrent lesions were localized and small, gynecological oncologists did not consider surgical treatment. Based on the patient’s genetic phenotype, we recommend immunotherapy with or without radiotherapy according to the guidelines. Re-irradiation needs are individualized according to the extent of disease, the time elapsed from the previous treatment, and prior radiation fields. In addition, recurrences less than 2-4 cm and recurrences with longer intervals of disease-free time tend to improve the prognosis. The most frequent re-irradiation method is intracavitary or interstitial BT (22). The patient refused immunotherapy because of possible side effects, and we only performed interstitial BT for the presacral metastatic lymph node based on the above treatment modalities and the previous permanent seed implant. We will be evaluating the therapeutic effect and long-term side effects over time.

## Conclusion

The present case report shows disease manifestations and outcomes of LS-EC. It indicates that EC-LS patients, carriers with combined EPCAM-MSH2 deficiency might experience better oncologic outcomes even with early recurrence. Further molecular/genetic studies are needed to evaluate prognostic factors in patients with combined EPCAM-MSH2 deletion LS-EC.

## Author Contributions

Acquisition of data: RH, DL, QW, and ZZ. Manuscript writing: RH, DL and XD. Critical review of the manuscript: All authors. All authors contributed to the article and approved the submitted version.

## Conflict of Interest

The authors declare that the research was conducted in the absence of any commercial or financial relationships that could be construed as a potential conflict of interest.

## Publisher’s Note

All claims expressed in this article are solely those of the authors and do not necessarily represent those of their affiliated organizations, or those of the publisher, the editors and the reviewers. Any product that may be evaluated in this article, or claim that may be made by its manufacturer, is not guaranteed or endorsed by the publisher.
